# 14-3-3θ Does Not Protect against Behavioral or Pathological Deficits in Alzheimer’s Disease Mouse Models

**DOI:** 10.1523/ENEURO.0368-21.2022

**Published:** 2022-06-22

**Authors:** Mary Gannon, Bing Wang, Sara Anne Stringfellow, Stephan Quintin, Itzel Mendoza, Thanushri Srikantha, A. Claire Roberts, Takashi Saito, Takaomi C. Saido, Erik D. Roberson, Talene A. Yacoubian

**Affiliations:** 1 Center for Neurodegeneration and Experimental Therapeutics, Department of Neurology, University of Alabama at Birmingham, Birmingham, AL 35294; 2 University of South Alabama College of Medicine, Mobile, AL 36688; 3 Medical Scientist Training Program, University of Florida, Gainesville, FL 32611; 4 RIKEN Center for Brain Science, Saitama 351-0198, Japan; 5 Department of Neurocognitive Science, Institute of Brain Science, Nagoya City University Graduates School of Medical Sciences, Nagaya 467-8601, Japan

**Keywords:** β-amyloid, 14-3-3, Alzheimer’s disease, cortex, hippocampus, mouse model

## Abstract

Alzheimer’s disease (AD) is characterized by progressive cognitive impairment associated with synaptic dysfunction and dendritic spine loss and the pathologic hallmarks of β-amyloid (Aβ) plaques and hyperphosphorylated tau tangles. 14-3-3 proteins are a highly conserved family of proteins whose functions include regulation of protein folding, neuronal architecture, and synaptic function. Additionally, 14-3-3s interact with both Aβ and tau, and reduced levels of 14-3-3s have been shown in the brains of AD patients and in AD mouse models. Here, we examine the neuroprotective potential of the 14-3-3θ isoform in AD models. We demonstrate that 14-3-3θ overexpression is protective and 14-3-3θ inhibition is detrimental against oligomeric Aβ-induced neuronal death in primary cortical cultures. Overexpression of 14-3-3θ using an adeno-associated viral (AAV) vector failed to improve performance on behavioral tests, improve Aβ pathology, or affect synaptic density in the J20 AD mouse model. Similarly, crossing a second AD mouse model, the *App^NL-G-F^* knock-in (APP KI) mouse, with 14-3-3θ transgenic mice failed to rescue behavioral deficits, reduce Aβ pathology, or impact synaptic density in the APP KI mouse model. 14-3-3θ is likely partially insolubilized in the APP models, as demonstrated by proteinase K digestion. These findings do not support increasing 14-3-3θ expression as a therapeutic approach for AD.

## Significance Statement

Despite being the most common form of neurodegeneration, effective treatments for Alzheimer’s disease (AD) remain elusive and therefore novel therapeutic targets are needed. 14-3-3 proteins, a highly conserved family of chaperone proteins involved in regulating synaptic function and neuronal architecture, are reduced in human AD patients, making them a promising novel target. In this study, we examined the effects of increasing the 14-3-3θ isoform in AD models. We observed that 14-3-3θ overexpression reduces oligomeric β-amyloid (Aβ) toxicity in cortical cultures. However, increasing the 14-3-3θ expression in two different AD mouse models failed to rescue behavioral or pathologic phenotypes, indicating a more complicated relationship between 14-3-3s and AD.

## Introduction

Alzheimer’s disease (AD) is the most common neurodegenerative disease, with progressive neuronal loss leading to gradual cognitive decline and ultimately death. With the anticipated rise in AD cases in the next several decades, the expected societal burden and financial costs of AD are skyrocketing, with predicted health care costs for AD reaching $1.2 trillion in 2050 (Alzheimer’s Association, 2014). In addition to neuronal loss in the hippocampus and frontotemporal cortex, AD is characterized pathologically by two major hallmarks, β-amyloid (Aβ) plaques and tau tangles. However, cognitive impairment correlates more strongly with synaptic dysfunction and spine loss than with plaque or tangle burden (DeKosky and Scheff, 1990; Terry et al., 1991; Hardy and Selkoe, 2002; Selkoe, 2011; Pozueta et al., 2013; Tu et al., 2014; Boros et al., 2017).

Several pieces of evidence point to a potential role for the 14-3-3 proteins in AD. 14-3-3 proteins are a highly conserved family of proteins that act via protein-protein interactions to regulate several cellular functions important to neuronal function (Dougherty and Morrison, 2004; Gehler et al., 2004; Mackintosh, 2004; Chen and Roche, 2009; Lee et al., 2009; Ramser et al., 2010; Yoon et al., 2012; Chung et al., 2015; Foote et al., 2015; Workman et al., 2015; Lavalley et al., 2016; Kaplan et al., 2017; Pennington et al., 2018). 14-3-3s interact with Aβ and tau and are found in Aβ plaques and neurofibrillary tangles in human AD brains (Hashiguchi et al., 2000; Liao et al., 2004; Umahara et al., 2004; Sumioka et al., 2005). Genetic variants in the 14-3-3 genes are associated with AD risk (Mateo et al., 2008a,b). 14-3-3s are important regulators of neuronal architecture: they stabilize synaptic spines and promote axonal and dendritic growth (Gohla and Bokoch, 2002; Gehler et al., 2004; Ramser et al., 2010; Yoon et al., 2012; Foote et al., 2015; Lavalley et al., 2016; Kaplan et al., 2017). Additionally, 14-3-3s regulate NMDA receptor (NMDAR) trafficking to the synapse and impact cerebellar and hippocampal long-term potentiation (Simsek-Duran et al., 2004; Chen and Roche, 2009; Qiao et al., 2014; Chung et al., 2015). Indeed, the functional 14-3-3 knock-out mice expressing the pan 14-3-3 inhibitor, difopein, show deficits mimicking several aspects of AD mouse models, including reduced spine density, reduced NMDAR subunits at the synapse, impaired long-term potentiation, and hippocampal-dependent learning and memory deficits (Qiao et al., 2014; Foote et al., 2015). Aged rats have reduced 14-3-3θ in hippocampal synapses, and lower 14-3-3θ levels in aged rats correlate with decreased cognitive function (VanGuilder et al., 2010, 2011). Recently, a dramatic 40% reduction in total soluble 14-3-3 protein levels in AD brains was observed, relative to age-matched controls, with a converse increase in total 14-3-3 levels in the insoluble fraction (McFerrin et al., 2017). This is consistent with recent data showing reduced solubility of 14-3-3s, including the 14-3-3θ isoform, in the APP-presenilin (APP/PS1) mouse model compared with controls (G. Xu et al., 2013).

Taken together, this evidence suggests that 14-3-3θ dysfunction contributes to cognitive deficits in AD. In this study, we used primary neuronal cultures and two AD mouse models, the J20 APP transgenic model and the *App^NL-G-F^* knock-in (APP KI) mouse, to test the impact of 14-3-3θ in AD. While we found that 14-3-3θ reduced Aβ-induced neuron loss in culture, 14-3-3θ failed to reduce Aβ plaque load, alter synaptic density, or improve cognitive deficits in the AD mouse models.

## Materials and Methods

### Mice

All mice used in these studies followed the guidelines of the National Institutes of Health (NIH) and University of Alabama at Birmingham Institutional Animal Care and Use Committee (IACUC), and all work performed was approved by University of Alabama at Birmingham IACUC. Equal numbers of male and female mice were used for all studies. Mice were group housed under 12/12 h light/dark cycle. The 14-3-3θ line used expressed human 14-3-3θ under the neuronal promoter Thy1.2 (Lavalley et al., 2016; Wang et al., 2018; Underwood et al., 2021). The difopein line used expressed difopein-enhanced yellow fluorescent protein (eYFP) under the promoter Thy1.2 and was initially obtained from Yi Zhou at Florida State University (Qiao et al., 2014). To obtain both transgenic and littermate controls for the primary culture experiments, both hemizygous 14-3-3θ and difopein mice were crossed separately with C57BL/6J mice from The Jackson Laboratory (catalog #000664; RRID: IMSR_JAX:000664). J20 transgenic mice carry both the APP Swedish (KM670/671NL) and APP Indiana (V717F) mutations and were on a C57BL6/J background (Mucke et al., 2000). Homozygous *App^NL-G-F/NL-G-F^* (APP KI) mice (Saito et al., 2014) were provided by the Riken BRC through the National Bio-Resource Project of the MEXT, Japan. Hemizygous 14-3-3θ transgenic mice (14-3-3θ tg) were crossed with homozygous APP KI mice (*App^NL-G-F/NL-G-F^*) to create *App^WT/NL-G-F^*/14-3-3θ tg and *App^WT/NL-G-F^*/WT. These mice were then crossed with each other to yield, among other genotypes, the desired *App^NL-G-F/NL-G-F^*/14-3-3θ tg and *App^NL-G-F/NL-G-F^*/WT mice. Crossing these two mice with each other produced the *App^NL-G-F/NL-G-F^*/14-3-3θ tg (APP KI/14-3-3θ) and *App^NL-G-F/NL-G-F^*/WT (APP KI) mice used in experiments, without the additional possible genotypes. Control mice (nTg) without either the mutant APP or the overexpressed 14-3-3θ were created by crossing a heterozygous APP KI mouse (*App^WT/NL-G-F^*) with a nTg C57BL/6 mouse.

### Adeno-associated virus (AAV) preparation and injection

AAV2/CBA-IRES2-eGFP-WPRE (AAV-GFP) and AAV2/CBA-14-3-3θ-V5-his-IRES-eGFP-WPRE (AAV-14-3-3θ/GFP) were constructed as previously described (St Martin et al., 2007; Ding et al., 2015). Male and female J20 transgenic mice and littermate controls were deeply anesthetized with 5% isoflurane and maintained at 0.25−4% during surgery. Mice were bilaterally injected with AAV at 8–11 weeks of age using a stereotactic setup into the dentate gyrus [anteroposterior (AP): − 2.0 from bregma; mediolateral (ML): ±1.3 from midline; dorsoventral (DV): − 2.2 below dura]. Mice were injected with 2 μl of either AAV-GFP (titer: 1.8E + 12 vg/ml, viral genomes/ml) or AAV-14-3-3θ/GFP (titer: 7.0E + 11 vg/ml) at a rate of 0.25 μl/min using a microinjection pump.

### Aβ oligomer (Aβo) preparation

Aβ (rPeptide, A-1163-2) was reconstituted in HFIP (Sigma-Aldrich, 105228) and aliquoted. After full evaporation of HFIP in fume hood, aliquots were stored at −80°C until ready for use. One day before experiments, an Aβ aliquot was resuspended to 100 μm. The Aβ was first resuspended in a small volume in sterile DMSO (Sigma D2650) by vortexing and brought to the final volume using HBSS (Invitrogen14,175–095). After pipette trituration, the Aβ was sonicated on high for 15 cycles of 45 s on, 15 s off. The Aβ was then left overnight at 4°C to form oligomers.

### Aβ toxicity assay

Hippocampal or cortical regions were dissected from male and female postnatal (P)0 mice and incubated at 37°C for 25 min in papain. Cells were then washed with neurobasal-A media containing B-27 supplement, Glutamax, and 5% FBS and then triturated using fire polished glass pipettes. Cells were pelleted by centrifugation at 700 rpm for 5 min and were resuspended in neurobasal-A media containing B-27 supplement, Glutamax, and 5% FBS and plated on 18 mm poly-d-lysine-coated coverslips. After 12–16 h, media were replaced by neurobasal-A media containing B-27 supplement, Glutamax, and Arabinose C at 6 μm. Fifty percent media changes were made every 7 d. After 14 d, 1 or 5 μm of Aβ oligomers (Aβo) or vehicle control were added. After 24 h of Aβo treatment, cells were incubated with ethidium homodimers to stain dead cells and Hoechst dye to stain all cells, and then mounted onto slides using VectaShield (Vector Laboratories). Total cells stained with both ethidium homodimers and Hoechst were then counted and a percentage of dead cells calculated.

### Behavior

All behavior tests were performed with approximately equal numbers of male and female mice.

#### Open field

Mice were placed in a 48 × 48-inch open arena with clear plexi-glass walls and allowed to freely move for 4 min while being recorded using EthoVision software. Video was then analyzed for overall velocity and distance moved across all planes (vertical and horizontal).

#### Elevated plus maze (EPM)

Mice were placed in the center of plus shaped maze elevated above the floor. Two oppositely placed arms of the maze are open and the remaining two arms are closed. Mice were allowed to freely explore the maze for 4 min while being recorded using EthoVision software. Video was then analyzed to calculate the percentage of time that mice spent in the open arm.

#### Morris water maze (MWM)

Mice were placed in a pool of water with a hidden platform submerged under the surface of the water. During the acquisition phase of the test, the time taken for the mice to find the platform (latency to platform) was recorded for four trials daily for 5 d. Immediately following the final trial on day 5 of the acquisition phase, a probe trial was conducted where the platform was removed. The percentage of time spent in the quadrant where the platform had been located (target quadrant) was then calculated. All trials were recorded and analyzed using EthoVision software.

#### Passive avoidance

The experiment was performed using a two-compartment box in which one chamber was darkened, and a grid floor of stainless-steel bars was connected to an internal shock source (Gemini II Avoidance System). On the training day, each mouse was placed in the lighted chamber with the interchamber door closed. After 60 s the door was opened and when the mouse crossed to the darkened side the door was closed and a 0.5 mA × 2 s foot shock was administered. Mice were left in the dark chamber for 30 s before being returned to their home cage. Mice were tested 24 h later and the protocol was repeated but without application of a foot-shock, with a cutoff time of 5 min. For both training and testing, the latency was measured as the time when all four paws entered into the darkened chamber.

### Immunohistochemistry

Immediately following the conclusion of behavior testing, mice were perfused with PBS before 4% paraformaldehyde using a forced pump system. The brains were then processed and embedded in paraffin and were sliced in 7-μm-thick sections. Sections were dewaxed and rehydrated followed by antigen retrieval done by autoclaving in citrate buffer. Sections were then quenched with 0.3% hydrogen peroxide in methanol before blocking in 5% NGS with 0.1% Triton X-100. Sections were incubated overnight with primary antibody [mouse GFP (abcam ab1218; RRID: AB_298911), rabbit V5 (Sigma V8137; RRID: AB_261889), rabbit NMDA receptor 2a (NR2A; Millipore 07–632; RRID: AB_310837), guinea pig vesicular glutamate transporter 1 (VGLUT1) (Synaptic Systems 135 304; RRID: AB_887878), mouse 14-3-3θ (Santa Cruz SC-69720; RRID: AB_2218224), or mouse HA (Biolegend 901501; RRID: AB_2565006)]. After washing with TBS, sections were incubated with Cy3-conjugated goat anti-mouse, Cy3-conjugated goat anti-rabbit, or Alexa Fluor 488 goat anti-guinea pig secondary antibody. Slides were coverslipped using ProLong Diamond Antifade mounting solution (Thermofisher Scientific) and imaged using an Olympus BX51 epifluorescence microscope or Nikon Eclipse Ti2 confocal microscope.

### Aβ plaque load quantification

Every fourth section through the hippocampus was stained for Aβ, using the TSA Plus amplification kit (Akoya Biosciences, NEL763001KT). Sections were de-waxed and rehydrated and antigen retrieval was done by autoclaving in citrate buffer. Sections were quenched with 0.3% hydrogen peroxide in methanol before blocking and overnight incubation with primary antibody (mouse anti-Aβ 82E1, IBL 10 326). After washing, sections were incubated with goat anti-mouse IgG biotinylated secondary antibody (Vector Laboratories, BA-9200). Sections were then incubated in HRP-conjugated streptavidin (Thermo Scientific) and finally with the FITC-conjugated Tyramide. Slides were coverslipped using ProLong Diamond Antifade mounting solution (Thermofisher Scientific) and imaged using an Olympus BX51 epifluorescence microscope. Images were then quantified using the ‘analyze particles’ function in ImageJ to calculate percent plaque load in both hippocampus and cortex.

### Synapsin quantification

To estimate synaptic density, paraffin embedded sections were stained for the presynaptic marker synapsin. Paraffin embedded sections were stained as described for the Aβ immunohistochemistry methods using a rabbit synapsin primary antibody (Invitrogen 51-5200), followed by the TSA Plus amplification kit as described above. 60× images of each hippocampal section were acquired using a Nikon Eclipse Ti2 confocal microscope. The area positive for synapsin staining was quantified using ImageJ software. Images were converted to 8 bit in ImageJ, and the threshold was set with a lower limit of 15 and upper limit of 255. The area fraction and total area of the image was obtained through the “measure” feature of ImageJ. Any blank spaces in the image because of tears in the tissue were then circled as ROIs and the total area of the empty space was obtained using the “measure” feature again. The empty space area was subtracted from the initial total area of the image, and the percent area positive for synapsin staining was calculated. For each mouse, two images from each of three to four hippocampal sections were analyzed and averaged.

### Proteinase K digestion

Sections were dewaxed and rehydrated and then treated for 10 min at 37°C with 20 μg/ml of proteinase K diluted in TBS or TBS only. Antigen retrieval was done by autoclaving in citrate buffer and sections were quenched with 0.3% hydrogen peroxide in methanol. Sections were then blocked in 5% NGS with 0.1% Triton X-100 and incubated overnight with primary antibody (mouse 14-3-3θ; Santa Cruz SC-69720; RRID: AB_2218224). After washing with TBS, sections were incubated with Cy3-conjugated goat anti-mouse secondary antibody. Slides were coverslipped using ProLong Diamond Antifade mounting solution (Thermofisher Scientific) and imaged using a Nikon Eclipse Ti2 confocal microscope.

### Statistical analysis

All statistical analyses and graphing were performed in Prism 9.2 (GraphPad Software, Inc., La Jolla, CA). Details of statistical tests are outlined in Table 1.

## Results

### 14-3-3 overexpression protects while inhibition exacerbates oligomeric Aβ-induced cell death *in vitro*

**Figure 1.**
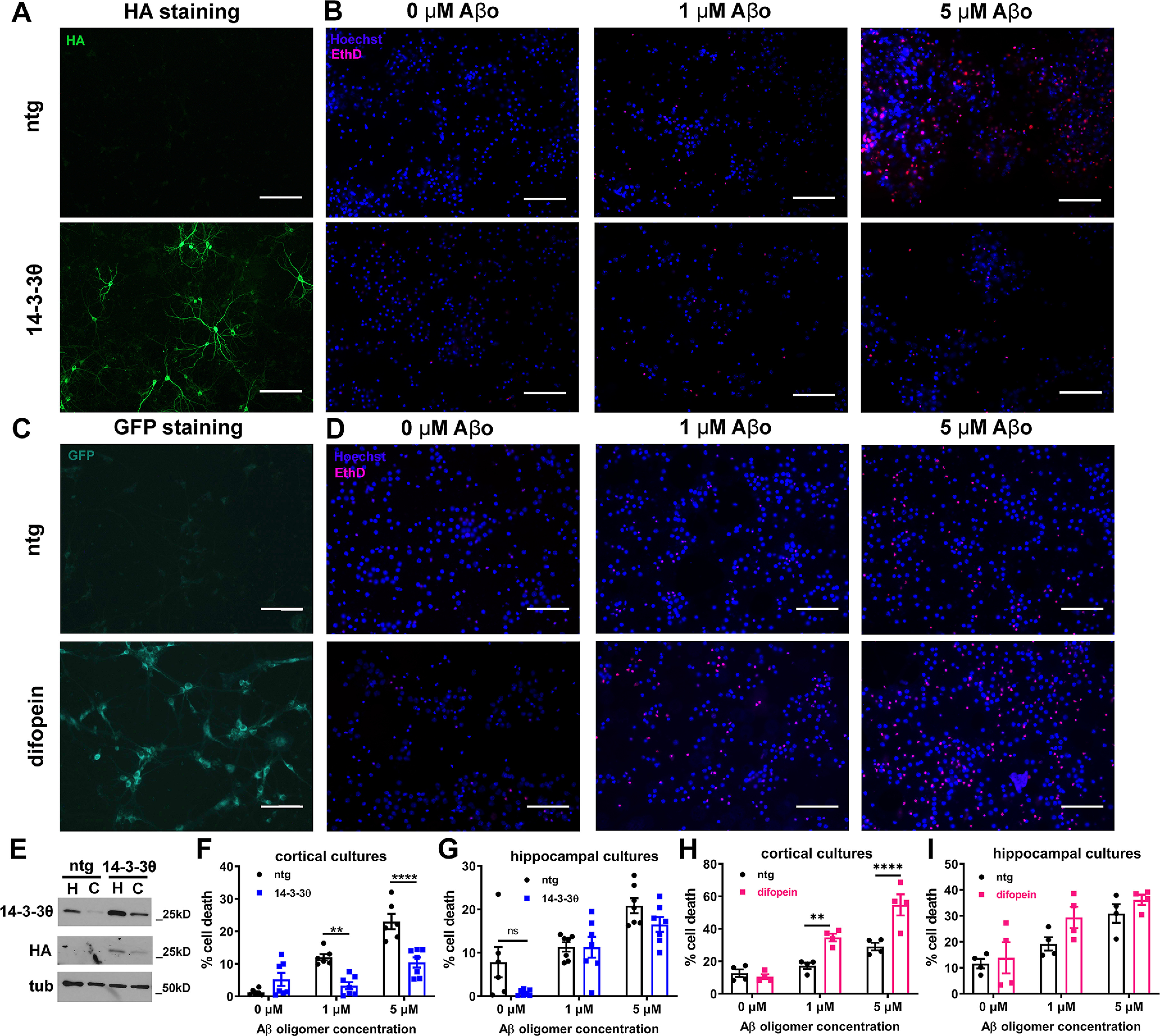
14-3-3θ reduces oligomeric Aβ toxicity in primary cortical cultures. ***A***, Immunocytochemistry for exogenous HA-tagged 14-3-3θ in nontransgenic (nTg) and 14-3-3θ transgenic mouse neuronal cultures. Scale bar: 100 μm. ***B***, Representative images of primary cortical neurons treated with oligomeric Aβ from nontransgenic or 14-3-3θ mice. Ethidium D (EthD) labels the nuclei of dying cells, while Hoechst 33342 stains the nuclei of all cells. Scale bar: 100 μm. ***C***, Immunocytochemistry for eYFP-difopein in nontransgenic and difopein transgenic mouse neuronal cultures. Scale bar: 100 μm. ***D***, Representative images of primary cortical neurons treated with oligomeric Aβ from nontransgenic or difopein mice. Ethidium D labels the nuclei of dying cells, while Hoechst 33342 stains the nuclei of all cells. Scale bar: 100 μm. ***E***, Western blotting for 14-3-3θ levels in Triton X-100 soluble fractions from hippocampal (H) and cortical (C) cultures from nontransgenic and 14-3-3θ mice. ***F***, Quantification of cell death in primary cortical neurons from nTg or 14-3-3θ littermate mice treated with Aβ oligomers for 24 h. *n* = 6 per condition; **p ≤* *0.01, ****p ≤* *0.0001 (Tukey’s multiple comparison test). Error bars represent standard error of the mean (SEM). ***G***, Quantification of cell death in primary hippocampal neurons from nTg or 14-3-3θ littermate mice treated with Aβ oligomers for 24 h. *n* = 7 per condition. Error bars represent SEM. ***H***, Quantification of cell death in primary cortical neurons from nTg or difopein littermate mice treated with Aβ oligomers for 24 h. *n* = 4 per condition, **p ≤* *0.01, ****p ≤* *0.0001 (Tukey’s multiple comparison test). Error bars represent SEM. ***I***, Quantification of cell death in primary hippocampal neurons from nTg or difopein littermate mice treated with Aβ oligomers for 24 h. *n* = 4 per condition. Error bars represent SEM.

We first examined the effects of 14-3-3s on oligomeric Aβ (Aβo) toxicity in neuronal cultures. We treated day *in vitro* (DIV)14 primary cortical neurons from 14-3-3θ transgenic mice, which demonstrate 14-3-3θ overexpression in the cortex and hippocampus (Fig. 1*A*,*E*; Underwood et al., 2021), and their nontransgenic (nTg) littermate controls with Aβo for 24 h. Aβo treatment induced neuronal toxicity in a dose-dependent manner in primary cortical neurons from nTg mice (Fig. 1*B*,*F*). At both 1 and 5 μm Aβ concentrations, we saw a reduction in Aβo-induced toxicity in 14-3-3θ cortical neurons compared with nTg neurons (two-way ANOVA: genotype *F*_(1,33)_ = 20.28, *p *<* *0.0001; Aβ dose *F*_(2,33)_ = 38.67, *p *<* *0.0001; interaction *F*_(2,33)_ = 15.40, *p *<* *0.0001; Fig. 1*B*,*F*). Interestingly, 14-3-3θ overexpression was minimally protective against Aβo toxicity in hippocampal cultures (two-way ANOVA: genotype *F*_(1,35)_ = 5.801, *p *=* *0.0214; Aβ dose *F*_(2,35)_ = 27.00, *p *<* *0.0001; interaction *F*_(2,35)_ = 1.626, *p *=* *0.2112; Fig. 1*G*).

To test the impact of 14-3-3 inhibition on Aβo toxicity, we used a functional knock-out mouse expressing the pan 14-3-3 inhibitor, difopein, tagged with eYFP under the Thy1.2 promoter. Difopein (dimeric fourteen-three-three peptide inhibitor) is a high-affinity 14-3-3 competitive antagonist peptide that disrupts 14-3-3/ligand interactions by binding within the amphipathic groove of all 14-3-3 isoforms (Masters et al., 2001). This mouse (line 132) expresses difopein-eYFP in neurons in the cortex and hippocampus (Fig. 1*C*; Qiao et al., 2014; Wang et al., 2018; Underwood et al., 2021). We treated DIV14 primary neurons from difopein mice with Aβo for 24 h. Cortical neurons from difopein mice showed an exacerbation of Aβo-induced toxicity in cortical neurons compared with nTg controls at both 1 and 5 μm (two-way ANOVA: genotype *F*_(1,18)_ = 25.85, *p *<* *0.0001; Aβ dose *F*_(2,18)_ = 42.68, *p *<* *0.0001; interaction *F*_(2,18)_ = 9.481, *p *=* *0.0015; Fig. 1*D*,*H*). However, difopein expression did not impact Aβo toxicity in hippocampal cultures (two-way ANOVA: genotype *F*_(1,18)_ = 3.952, *p *=* *0.0622; Aβ dose *F*_(2,18)_ = 16.47, *p *<* *0.0001; interaction *F*_(2,18)_ = 0.6126, *p *=* *0.5529; Fig. 1*I*). Taken together, these data show that 14-3-3θ is protective against Aβo-induced toxicity in cortical but not hippocampal neurons in culture.

### 14-3-3θ does not reduce cognitive decline in AD mouse models

We next examined the effects of 14-3-3θ overexpression in two different AD mouse models: (1) the J20 APP mouse (Mucke et al., 2000), and (2) the *App^NL-G-F^* knock-in (APP KI) mouse (Saito et al., 2014). The J20 APP mouse model expresses human APP with the Swedish (K670N, M671L) and Indiana (V717F) mutations under the PDGFβ promoter and demonstrates memory deficits and Aβ plaque deposition (Mucke et al., 2000; Chin et al., 2004, 2005). The newer APP KI model expressing human APP carrying the Swedish, Beyreuther/Iberian (I716F), and Arctic (E22G) mutations also demonstrates memory deficits and Aβ plaque deposition (Saito et al., 2014; Masuda et al., 2016).

**Figure 2.**
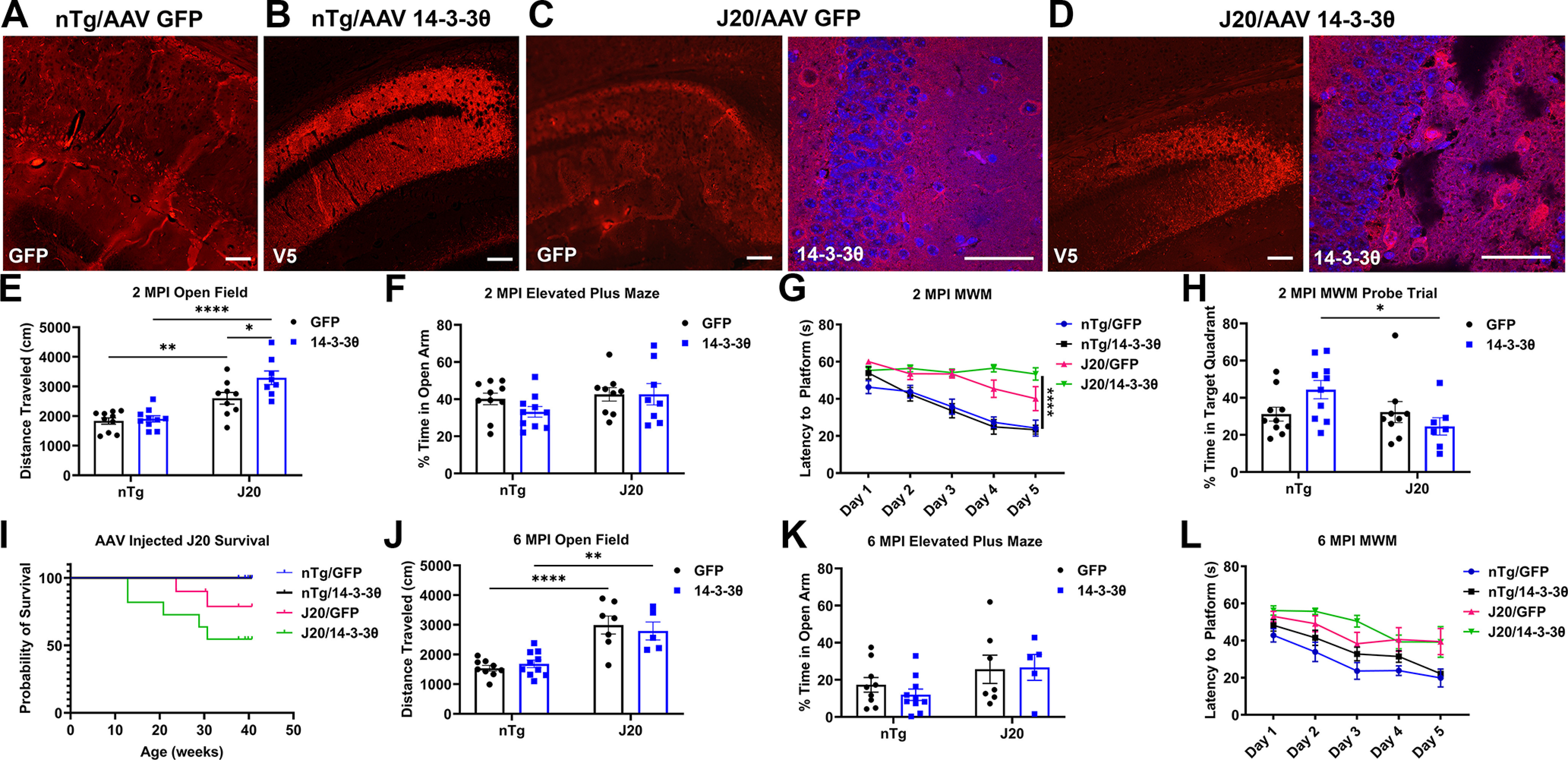
14-3-3θ overexpression does not rescue behavioral deficits in J20 mice. ***A***, Representative image of GFP immunostaining of an AAV-GFP-injected nontransgenic (nTg) mouse, indicating correct targeting and expression of the injected virus in the hippocampus of the mice. Scale bar: 100 μm. ***B***, Representative image of V5 immunostaining of an AAV-V5-tagged 14-3-3θ-injected nontransgenic mouse, indicating correct targeting and expression of the injected virus in the hippocampus of the mice. Scale bar: 100 μm. ***C***, Representative image of GFP and 14-3-3θ immunostaining of an AAV-GFP-injected J20 mouse. 14-3-3θ immunostaining detects the endogenous 14-3-3θ expressed in these mice. Scale bar: 100 μm for GFP image and 50 μm for 14-3-3θ image. ***D***, Representative image of V5 and 14-3-3θ immunostaining of an AAV-V5-tagged 14-3-3θ-injected J20 mouse. 14-3-3θ immunostaining detects the endogenous and exogenous 14-3-3θ expressed in these mice, while V5 immunostaining detects only the exogenous V5-tagged 14-3-3θ. Scale bar: 100 μm for GFP image and 50 μm for 14-3-3θ image. ***E***, Quantification of distance traveled in the open field task at 2 MPI. *n* = 8–10 mice per group; *p ≤* *0.05, **p ≤* *0.01, ****p ≤* *0.0001 (Tukey’s multiple comparison test). Error bars represent SEM. ***F***, Quantification of time spent in the open arm on the EPM at 2 MPI. *n* = 8–10 mice per group. Error bars represent SEM. ***G***, Quantification of latency to platform on the MWM at 2 MPI. *n* = 7–10 mice per group; ****p <* *0.0001 (Tukey’s multiple comparison test). Error bars represent SEM. ***H***, Quantification of the probe trial in the MWM at 2 MPI. *n* = 7–10 mice per group; *p ≤* *0.05 (Tukey’s multiple comparison test). Error bars represent SEM. ***I***, Quantification of mortality for GFP and 14-3-3θ-injected nTg and J20 mice. ***J***, Quantification of distance traveled in the open field task at 6 MPI. *n* = 5–10 mice per group; **p ≤* *0.01, ****p ≤* *0.0001 (Tukey’s multiple comparison test). Error bars represent SEM. ***K***, Quantification of time spent in the open arm on the EPM at 6 MPI. *n* = 5–10 mice per group. Error bars represent SEM. ***L***, Quantification of latency to platform on the MWM at 6 MPI. *n* = 5–10 mice per group. Error bars represent SEM.

We used both viral-mediated overexpression and transgenic means to test the impact of 14-3-3θ overexpression *in vivo*. We first used stereotactic injections of an AAV vector to overexpress 14-3-3θ in the hippocampi of nTg and J20 mice. Male and female 8- to 11-week-old mice were injected with either AAV-GFP or AAV-14-3-3θ/GFP into the hippocampi bilaterally. We confirmed expression of GFP or V5-tagged 14-3-3θ in the hippocampi of AAV-injected mice at the immediate conclusion of the behavioral experiments, approximately six months after viral injection (Fig. 2*A–D*). At two and six months after viral injection, we performed behavioral tests, including the open field, EPM, and MWM tests. The J20 mice have been previously shown to demonstrate behavioral alterations in these tests with increased exploratory movement on the open field test, altered anxiety on the EPM, and impaired memory on the MWM (Chin et al., 2005; Kobayashi and Chen, 2005; Ognibene et al., 2005; Cheng et al., 2007; Roberson et al., 2007; Meilandt et al., 2009; Harris et al., 2010).

On open field testing at two months postinjection (MPI) of the virus, when the mice were four to five months old, J20 mice showed more exploratory behavior than nTg mice (Fig. 2*E*, greater distance traveled), as previously described (Chin et al., 2005; Kobayashi and Chen, 2005; Ognibene et al., 2005; Cheng et al., 2007; Roberson et al., 2007; Meilandt et al., 2009; Harris et al., 2010), while the 14-3-3θ-injected J20 mice traveled significantly further than the GFP-injected J20 mice (two-way ANOVA: genotype *F*_(1,33)_ = 45.57, *p *<* *0.001; virus *F*_(1,33)_ = 5.910, *p *= 0.0207; interaction *F*_(1,33)_ = 3.746, *p *=* *0.0615; Fig. 2*E*). There was no difference between any of the groups in the EPM, indicating no difference in levels of anxiety at 2 MPI (two-way ANOVA: genotype *F*_(1,33)_ = 2.451, *p *=* *0.1270; virus *F*_(1,33)_ = 0.8338, *p *=* *0.3678; interaction *F*_(1,33)_ = 0.8565, *p *=* *0.3614; Fig. 2*F*). The J20 mice, regardless of type of virus injection, were impaired in the MWM task at 2 MPI, showing less ability to learn the platform location over the course of training (three-way ANOVA: genotype *F*_(1,85)_ = 105, *p *<* *0.0001; time *F*_(4,85)_ = 16.33, *p *<* *0.0001; virus *F*_(1,79)_ = 2.544, *p *=* *0.1147; time × genotype *F*_(4,85)_ = 3.84, *p *=* *0.0064; time × virus *F*_(4,79)_ = 0.7289, *p *=* *0.5749; genotype × virus *F*_(1,79)_ = 2.266, *p *=* *0.1362; time × genotype × virus *F*_(4,79)_ = 2.713, *p *=* *0.0357; Fig. 2*G*). On the final testing day, the 14-3-3θ-injected J20 mice were significantly slower at finding the platform than either nTg group (Tukey’s *post hoc* test: *p *<* *0.0001 for J20 14-3-3θ vs nTg GFP and for J20 14-3-3θ vs nTg 14-3-3θ), but there was no statistically significant difference between the GFP-injected J20 and the 14-3-3θ-injected J20 groups (Tukey’s *post hoc* test: *p *=* *0.5494). During the probe trial, the 14-3-3θ-injected J20 mice performed worse than the 14-3-3θ-injected nTg mice, but no other comparisons were statistically significant (two-way ANOVA: genotype *F*_(1,32)_ = 3.675, *p *=* *0.0642; virus *F*_(1,32)_ = 3.170, *p *=* *0.5773; interaction *F*_(1,32)_ = 4.622, *p *=* *0.0392; Fig. 2*H*).

Behavioral tests were repeated on the same cohort of mice at six months after AAV injection, when the mice were eight to nine months old, though the sample sizes for these later behavioral tests were limited because of J20 mouse mortality. While not statistically significant, the 14-3-3θ-injected J20 mice showed a higher mortality rate than the GFP-injected J20 mice (*p *=* *0.201; log-rank/Mantel–Cox test; Fig. 2*I*). Premature mortality rates around 15% have been described in the J20 mouse line (Palop et al., 2007). We observed that 2 out of 10 (20%) GFP-injected J20 mice died compared with 5 out of 11 (45%) 14-3-3θ-injected J20 mice by the conclusion of the experiment (Fig. 2*I*).

Similar to the earlier time point, the J20 mice showed greater distance moved in the open field test 6 MPI, regardless of type of virus injection (two-way ANOVA: genotype *F*_(1,27)_ = 40.84, *p *<* *0.0001; virus *F*_(1,27)_ = 0.1557, *p *=* *0.9016; interaction *F*_(1,27)_ = 0.7530, *p *=* *0.3932; Fig. 2*J*). While the J20 genotype was associated with increased time in the open arm of the EPM, indicating decreased anxiety in these mice, there was no statistical difference between GFP-injected J20 mice and 14-3-3θ-injected J20 mice (two-way ANOVA: genotype *F*_(1,27)_ = 4.821, *p *=* *0.0369; virus *F*_(1,27)_ = 0.1706, *p *=* *0.6828; interaction *F*_(1,27)_ = 0.3644, *p *=* *0.5511; Fig. 2*K*). On the MWM, the J20 mice did significantly worse than nTg mice at learning the location of the platform, but there were no differences between 14-3-3θ-injected J20 mice and GFP-injected J20 mice on the final day of testing (three-way ANOVA: genotype *F*_(1,75)_ = 46.69, *p *<* *0.0001; time *F*_(4,75)_ = 12.86, *p *<* *0.0001; virus *F*_(1,60)_ = 6.839, *p *=* *0.0113; time × genotype *F*_(4,75)_ = 0.5912, *p *=* *0.67; time × virus *F*_(4,60)_ = 0.6958, *p *=* *0.5978; genotype × virus *F*_(1,60)_ = 0.3838, *p *=* *0.5379; time × genotype × virus *F*_(4,60)_ = 0.2333, *p *=* *0.9186; Fig. 2*L*). Based on these findings, we concluded that AAV-mediated overexpression of 14-3-3θ expression into the hippocampus was not protective in terms of behavioral decline.

**Figure 3.**
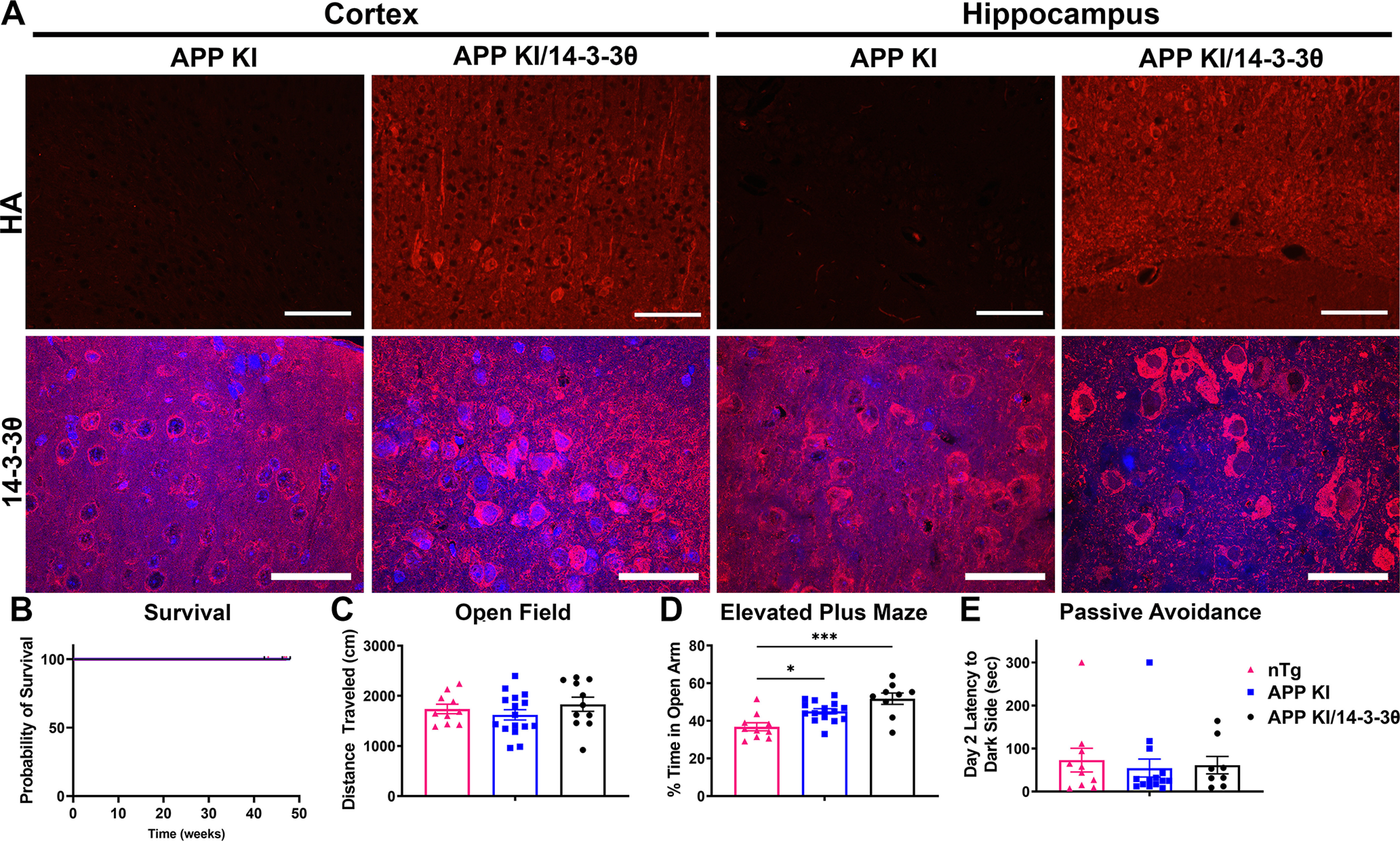
14-3-3θ overexpression does not modify behavior in APP KI mice. ***A***, Representative images of HA immunostaining in the cortex and hippocampus of APP KI mice demonstrates expression of HA-tagged 14-3-3θ in 14-3-3θ-overexpressing mice (APP KI/14-3-3θ) but not mice without overexpression (APP KI). 14-3-3θ immunostaining detects both endogenous and exogenous 14-3-3θ expressed in APP KI and APP KI/14-3-3θ mice. Scale bar: 100 μm for HA images and 50 μm for 14-3-3θ images. ***B***, Quantification of mortality for nontransgenic, APP KI, and APP KI/14-3-3θ mice. ***C***, Quantification of distance traveled in the open field task at eight to nine months of age. *n* = 10–16 mice per group. Error bars represent SEM. ***D***, Quantification of time spent in the open arm on the EPM at eight to nine months of age. *n* = 10–15 mice per group; *p ≤* *0.05, ***p ≤* *0.001 (Tukey’s multiple comparison test). Error bars represent SEM. ***E***, Quantification of latency in crossing to the dark side on day 2 of the passive avoidance task. *n* = 8–14 mice per group. Error bars represent SEM.

We also examined the effect of 14-3-3θ in the APP KI model by crossing 14-3-3θ transgenic mice with APP KI mice. Unlike the J20 mice, we did not observe any mortality of these mice by the end of the experiment (Fig. 3*B*). As APP KI mice demonstrate much more subtle cognitive changes at later time points, we tested these mice at eight to nine months by open field, elevated zero maze, and the passive avoidance test (Masuda et al., 2016; Saito et al., 2014). The APP KI mice do not demonstrate deficits on the MWM, and thus we did not perform this test (Saito et al., 2014). We examined nTg (*App^WT/WT^*/WT), APP KI (*App^NL-G-F/NL-G-F^*/WT), and APP KI/14-3-3θ (*App^NL-G-F/NL-G-F^*/14-3-3θ tg) mice on these behavioral tests. After the behavior was concluded and the mice were killed, we confirmed exogenous HA-tagged 14-3-3θ expression in the cortex and hippocampus of APP KI/14-3-3θ mice, which was not observed in APP KI mice (Fig. 3*A*). NTg, APP KI, and APP KI/14-3-3θ mice showed no difference in distance traveled in the open field (one-way ANOVA: *F*_(2,34)_ = 0.9122, *p *= 0.4112; Fig. 3*C*). APP KI and APP KI/14-3-3θ mice spent more time in the open arm in the elevated zero maze compared with the nTg mice, indicating decreased anxiety, but there was no difference between APP KI and APP KI/14-3-3θ mice (one-way ANOVA: *F*_(2,31)_ = 11.17, *p *=* *0.0002; Fig. 3*D*). On the passive avoidance test, none of the three groups showed any delay in crossing to the dark side of the shuttle box on day 2, and there was no difference between the groups (one-way ANOVA: *F*_(2,29)_ = 0.1705, *p *=* *0.8441; Fig. 3*E*). Of note, the passive avoidance test may have been impacted by an unexpected and uncontrollable alarm that randomly went off in the animal facility during housing of the APP KI cohort. As with the J20 mice, 14-3-3θ overexpression did not rescue behavioral changes in the APP KI mice.

### 14-3-3θ does not affect Aβ plaque deposition *in vivo*

**Figure 4.**
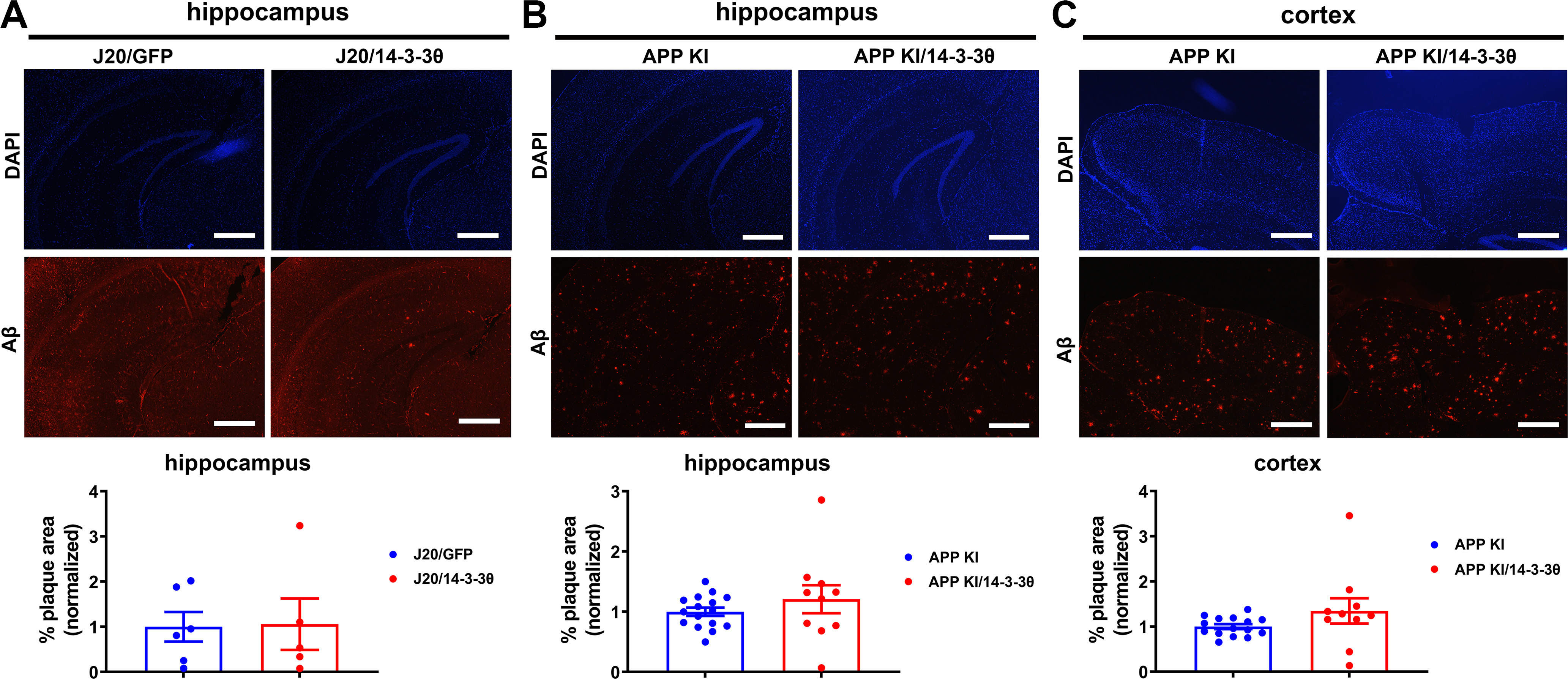
14-3-3θ overexpression does not reduce Aβ plaque burden in AD mice. ***A***, Representative hippocampal images and quantification of total plaque area of J20 mice injected with either AAV-GFP or AAV-14-3-3θ at 6 MPI. *n* = 5–6 per group. Error bars represent SEM. Scale bar: 500 μm. ***B***, Representative hippocampal images and quantification of total plaque area of APP KI and APP KI/14-3-3θ mice at eight to nine months of age. *n* = 10–16 per group. Error bars represent SEM. Scale bar: 500 μm. ***C***, Representative cortical images and quantification of total plaque area of APP KI and APP KI/14-3-3θ mice at eight to nine months of age. *n* = 10–15 per group. Error bars represent SEM. Scale bar: 500 μm.

Immediately on the conclusion of the last behavioral time point, both cohorts of mice were killed, and their brains were examined for Aβ plaque load using immunofluorescent staining. As expected, the J20 mice had Aβ plaques in the hippocampus, whereas Aβ plaques were not observed in the nTg controls (Fig. 4*A*). There was no difference in hippocampal Aβ plaque load between the GFP-injected and 14-3-3θ-injected J20 mice (unpaired, two-tailed *t* test: *t*_(9)_ = 0.09254, *p *=* *0.9238; Fig. 4*A*). We did not analyze plaque load in the cortex of J20 mice as the AAV injection was directed to the hippocampi. Similarly, the APP KI mice had a large number of plaques in both the hippocampus and cortex, but there was no difference between the APP KI and APP KI/14-3-3θ in either the hippocampus (unpaired, two-tailed *t* test: *t*_(24)_ = 1.038, *p *=* *0.3084; Fig. 4*B*) or cortex (unpaired, two-tailed *t* test: *t*_(23)_ = 1.475, *p *=* *0.1538; Fig. 4*C*). Based on these findings, 14-3-3θ overexpression did not reduce Aβ plaque burden in either the J20 or APP KI mouse models.

### 14-3-3θ does not affect synaptic density in APP models *in vivo*

**Figure 5.**
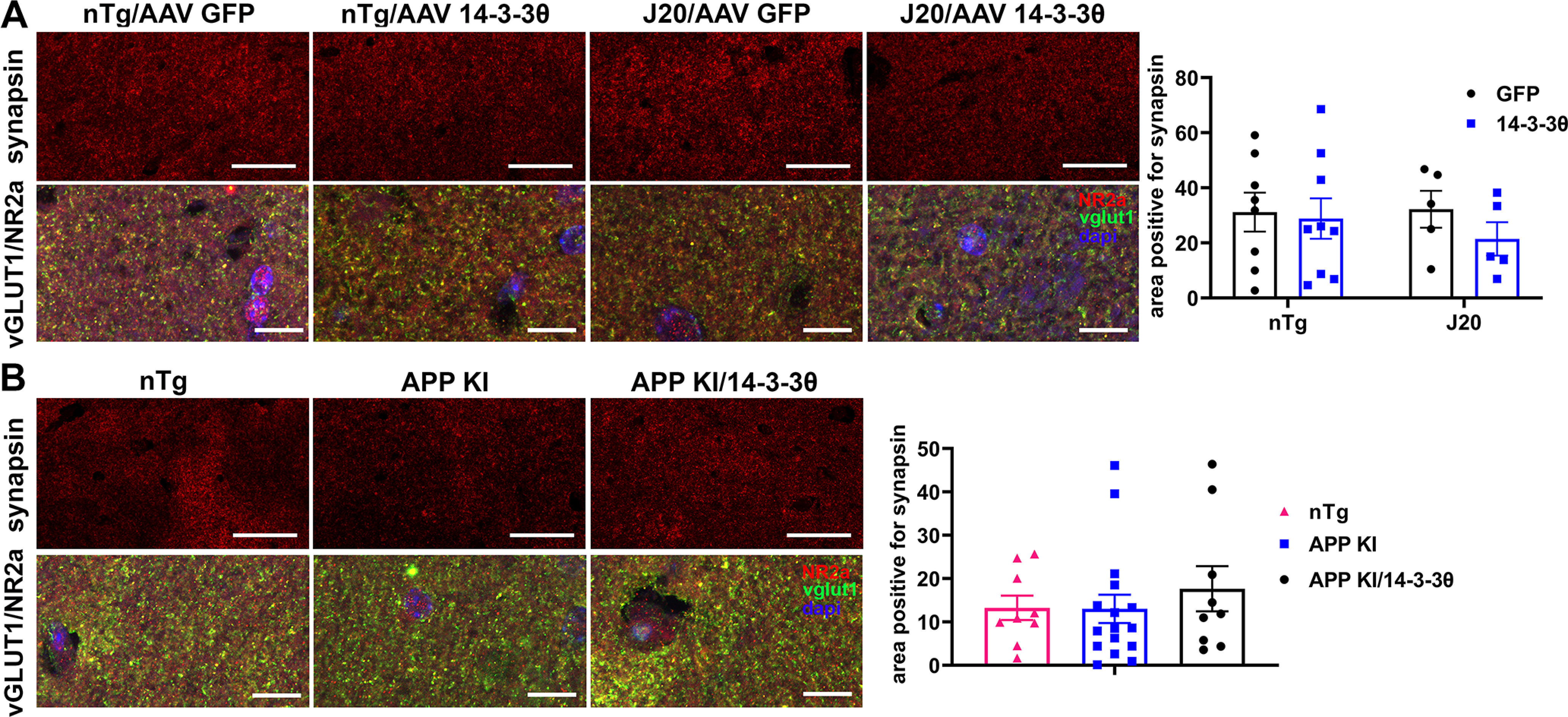
14-3-3θ overexpression does not impact synaptic density in AD mice. ***A***, Representative hippocampal images of synapsin, vGLUT1, and NR2A immunoreactivity and quantification of synapsin immunoreactivity in nontransgenic and J20 mice injected with either AAV-GFP or AAV-14-3-3θ at 6 MPI. *n* = 5–9 per group. Error bars represent SEM. Scale bars: 50 μm for synapsin images and 10 μm for vGLUT1/NR2A images. ***B***, Representative hippocampal images of synapsin, vGLUT1, and NR2A immunoreactivity and quantification of synapsin immunoreactivity in nontransgenic, APP KI, and APP KI/14-3-3θ mice at eight to nine months of age. *n* = 9–16 per group. Error bars represent SEM. Scale bars: 50 μm for synapsin images and 10 μm for vGLUT1/NR2A images.

We also examined mouse brains for potential loss of synaptic density by staining for the presynaptic marker synapsin in both cohorts of mice. In the J20 mice, there was no difference in the area that stained positive for synapsin, regardless of genotype or injection type (two-way ANOVA: genotype *F*_(1,23)_ = 0.1737, *p *=* *0.6807; virus *F*_(1,23)_ = 0.7492, *p *=* *0.3957; interaction *F*_(1,23)_ = 0.3085, *p *=* *0.5839; Fig. 5*A*). Similarly, the APP KI mice showed no changes in synapsin, regardless of genotype (one-way ANOVA: *F*_(2,31)_ = 0.4169, *p *=* *0.6627; Fig. 5*B*). Immunostaining with the presynaptic marker vesicular glutamate transporter vGLUT1 and postsynaptic marker NR2A confirmed synaptic integrity in the brains of both mouse cohorts (Fig. 5). Based on these results, we concluded that 14-3-3θ overexpression did not alter synaptic density in either cohort of mice.

### APP KI mice show altered subcellular 14-3-3θ distribution and solubility

**Figure 6.**
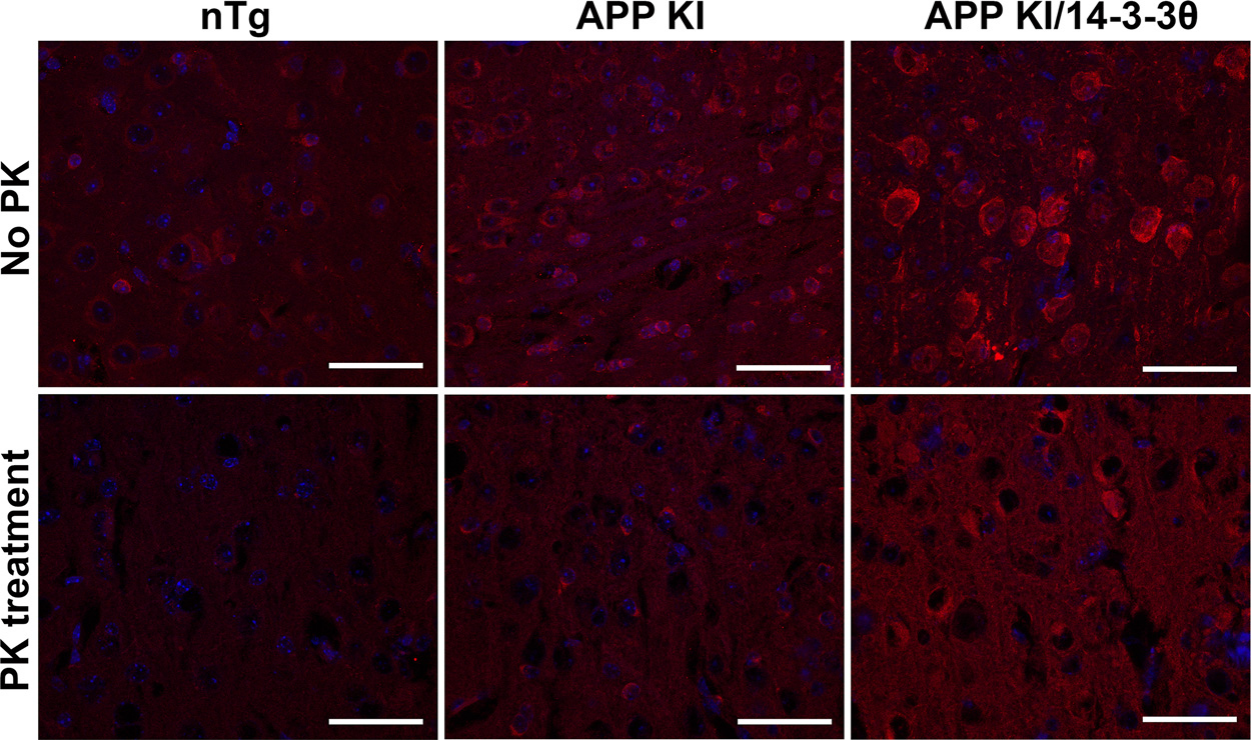
14-3-3θ immunoreactivity is partially proteinase K-resistant in APP KI mice. Representative images of 14-3-3θ immunostaining in cortical slices with and without proteinase K (PK) treatment from nontransgenic, APP KI, and APP KI/14-3-3θ mice. Scale bar: 50 μm.

Given the lack of protective effect for 14-3-3θ overexpression in the APP models, we examined the subcellular 14-3-3θ distribution in J20 and APP KI mice. In GFP-injected J20 mice and in APP KI mice, 14-3-3θ immunoreactivity was distributed in a very intense rim within the cytoplasm of neurons in deep cortical neurons and hippocampal neurons compared with that seen in nTg mice (Figs. 2*C*, 3*A*, 6). A similar pattern was observed in the presence of 14-3-3θ overexpression in 14-3-3θ-injected J20 mice and in the APP KI/14-3-3θ mice (Figs. 2*D*, 3*A*, 6). This pattern of staining in some neurons suggested possible insolubilization of 14-3-3θ in J20 and APP KI mice, as has been observed in human AD brains and in AD mouse models (G. Xu et al., 2013; McFerrin et al., 2017). To test for insoluble 14-3-3θ, brain sections were treated with proteinase K. While proteinase K digestion eliminated 14-3-3θ immunoreactivity in nTg mice, 14-3-3θ immunoreactivity, though reduced, was still observed in APP KI and APP KI/14-3-3θ mice with proteinase K digestion (Fig. 6).

## Discussion

In this study, we sought to study the effect of 14-3-3θ in AD, using both primary cultured neurons and *in vivo* AD mouse models. In primary culture, we found that 14-3-3θ overexpression ameliorated, while pan 14-3-3 inhibition exacerbated, Aβ toxicity in cortical cultures. No clear effect was observed in hippocampal cultures, pointing to a potential regional effect of 14-3-3 modulation. Based on these data, we hypothesized that overexpressing 14-3-3θ in AD mice would lead to an improvement in both the cognitive deficits and pathology. However, 14-3-3θ overexpression failed to reduce cognitive decline or Aβ pathology or affect synaptic density in either the J20 or APP KI models. In the case of the J20 mice, we injected AAV-14-3-3θ into the hippocampus because of the difficulty of obtaining widespread cortical AAV expression. Based on our *in vitro* data, targeting the hippocampus would not necessarily be sufficient to rescue behavior or pathology. However, boosting 14-3-3θ expression in both the cortex and the hippocampus, as was done in our APP KI × 14-3-3θ transgenic cross, was also not sufficient to rescue behavioral or pathologic changes in the cortex or hippocampus.

While it was unexpected to see 14-3-3θ protection in culture but not *in vivo*, this discrepancy likely reflects the vast differences in the environments of *in vitro* and *in vivo* experiments. In the *in vivo* system, other brain cell types, such as glia and astrocytes, interact with the neuronal population and could affect the findings. Alternatively, 14-3-3θ may be protective only in the short term, such that the protective effects were observed only in culture but not *in vivo* at the time points examined. Additionally, our *in vitro* studies showed that the protective effect was limited to cortical cultures and not to hippocampal cultures. This finding suggests that any protective effect of 14-3-3θ could be restricted to certain neuronal populations such that any global protection may not be seen *in vivo*. Further, we propose that 14-3-3θ overexpression failed to protect against Aβ toxicity *in vivo* because of potential insolubilization of 14-3-3θ. We found altered patterns of 14-3-3θ immunoreactivity in both the APP KI mice and the APP KI/14-3-3θ mice, in which neurons showed intense, “rim-like” cytoplasmic staining in neurons in the cortex and hippocampus, compared with less intense, more diffuse immunoreactivity in nTg control mice. This immunoreactivity in both APP KI mice and the APP KI/14-3-3θ mice was partially resistant to proteinase K digestion, suggesting a reduction in the solubility of 14-3-3θ, even in the presence of 14-3-3θ overexpression. We hypothesize that the presence of the APP mutations since conception *in vivo* may drive the insolubilization and consequent dysfunction of 14-3-3θ and thus block its protective effect, even in the presence of overexpression. In contrast, *in vitro* Aβ oligomers are added to neurons cultured from 14-3-3θ mice that do not contain the APP mutations so that 14-3-3θ is soluble at the time of Aβo treatment, as demonstrated by Western blotting for 14-3-3θ using Triton X-100 soluble fractions from primary neurons (Fig. 1*C*). Because the neurons are only exposed to the Aβ oligomers for a relatively short time, it is likely not long enough to lead to the insolubility of 14-3-3θ; and therefore, 14-3-3θ is able to maintain its protective functions.

In the J20 mice, we examined cognitive function using the MWM. At both 2 and 6 MPI, the J20 mice showed a reduced ability to locate the platform, but there was no significance difference between J20 mice who received the AAV-GFP injection compared with the AAV-14-3-3θ/GFP. At the earlier time point of 2 MPI, the 14-3-3θ-injected J20 mice actually showed a trend of doing worse than the GFP-injected J20 mice. While no difference in water maze performance was noted at the 6 MPI time point between GFP-injected and 14-3-3θ-injected J20 mice, increased mortality of the 14-3-3θ-injected J20 mice by 6 MPI could have limited our ability to detect a small difference. However, we did not see an increase in mortality in the APP KI/14-3-3θ, which were similarly impaired compared with APP KI mice, so it is unlikely that our lack of improvement was due solely to the poor health or increased mortality of the 14-3-3θ-injected J20 mice. 14-3-3θ showed no protection against cognitive decline and potentially worsened cognitive function and mortality in the J20 model.

In the APP KI mice, we assessed cognitive function by using the passive avoidance task. Surprisingly, we failed to see the expected differences in latency to cross to the dark side in APP KI mice compared with nTg controls. Previous studies have shown that APP KI mice perform poorly in this task compared with nTg control mice (Masuda et al., 2016; Zhang et al., 2020). One possible explanation for the lack of an effect of the APP KI mice in this behavioral test could be a result of early life exposure to intermittent stress. Unfortunately, because of circumstances out of our control, these mice were exposed to intermittent stress in the form of a randomly malfunctioning fire alarm in the animal facility where the mice were housed. This early exposure to intermittent stress may have led to a desensitization to stress and thus a failure to respond in the passive avoidance task. Therefore, interpretation of this behavioral test is limited. The APP KI mice did demonstrate decreased anxiety as demonstrated by the EPM, as previously shown (Sakakibara et al., 2018; Zhang et al., 2020; Degawa et al., 2021), which was not changed by 14-3-3θ overexpression.

One surprising result of our study was a possible trend toward higher mortality of the 14-3-3θ-injected J20 mice compared with the GFP-injected mice. A total of 45% (5/11) of the 14-3-3θ-injected mice died before our planned kill date, whereas only 20% (2/10) of the GFP-injected mice failed to survive. Of note, we had previously noted a high mortality rate of 14-3-3θ/J20 double transgenic mice when we tried to cross the 14-3-3θ transgenic mice with the J20 mice as an initial experimental approach. Because of the early mortality of double transgenic mice from this cross, we decided to switch to the AAV approach. One potential cause for this increased death within the 14-3-3θ-overexpressing J20 mice could be seizures. The J20 mouse line, like most lines expressing high levels of Aβ, has hippocampal hyperexcitability that leads to nonconvulsive seizures and higher rates of premature mortality (Palop et al., 2007). It is possible that the overexpression of 14-3-3θ increased seizure activity. Several studies have implicated 14-3-3s in seizure activity, although most of the data has pointed to a protective effect of other 14-3-3 isoforms in seizures (Schindler et al., 2006; Murphy et al., 2008; Brennan et al., 2013). Reducing tau in J20 mice reduces the severity of seizure activity in these mice (Palop et al., 2007), and 14-3-3ζ has also been shown to interact with tau and regulate tau aggregation *in vivo* (Sadik et al., 2009a,b). Therefore, it is possible that 14-3-3θ may interact with tau to affect seizure activity in the J20 mice. Finally, 14-3-3 proteins affect both neuronal morphology and glutamatergic receptor trafficking (Gohla and Bokoch, 2002; Gehler et al., 2004; Simsek-Duran et al., 2004; Chen and Roche, 2009; Ramser et al., 2010; Yoon et al., 2012; Qiao et al., 2014; Chung et al., 2015; Foote et al., 2015; Lavalley et al., 2016; Kaplan et al., 2017), such that any 14-3-3θ-induced alterations in receptor trafficking or neuronal morphology could impact seizure activity and thus mortality.

Beyond the lack of behavioral rescue by 14-3-3θ overexpression, we also failed to see any protection against pathologic changes. 14-3-3θ overexpression failed to reduce Aβ plaque deposition. We also measured synaptic density by staining for synapsin, but failed to see any significant effect of 14-3-3θ overexpression on synapsin immunoreactivity. While others have described a loss of synaptophysin immunoreactivity in the J20 mice (Chin et al., 2005), we did not detect a reduction for synapsin staining in J20 or APP KI mice compared with nTg mice by our approach. Our methodology was not optimal for measuring synaptic loss, as our brain sections were much thinner compared with those brain sections used in studies demonstrating synaptophysin changes in J20 mice. Golgi staining or super-resolution structured illumination microscopy would have been a more sensitive approach for measuring synapse loss (Hong et al., 2016). To our knowledge, quantification of synaptic loss has not been demonstrated in the APP KI mouse, although regional synaptophysin decrease around Aβ plaques has been seen (Saito et al., 2014).

14-3-3s are key interactors of several proteins implicated in other neurodegenerative diseases (Ostrerova et al., 1999; Hashiguchi et al., 2000; J. Xu et al., 2002; Sumioka et al., 2005; Kaltenbach et al., 2007; Nichols et al., 2010). A key role for 14-3-3θ dysfunction in Parkinson’s disease (PD) and dementia with Lewy bodies (DLB), both disorders marked by α-synuclein (αsyn) aggregation, has previously been established. A reduction of the 14-3-3θ isoform in cellular and mouse αsyn models and in human DLB brains has been shown (Yacoubian et al., 2010; Ding et al., 2013; McFerrin et al., 2017). 14-3-3θ overexpression reduces toxicity in multiple PD models, including neurotoxin, αsyn, and LRRK2 models (Yacoubian et al., 2010; Slone et al., 2011; Ding et al., 2015; Lavalley et al., 2016; Wang et al., 2018). In particular, 14-3-3θ overexpression reduces αsyn aggregation, cell-to-cell transmission, behavioral deficits, and neuronal loss in both *in vitro* and *in vivo* αsyn models (Wang et al., 2018; Underwood et al., 2021). This protective effect of 14-3-3θ in αsyn models is a result of its chaperone activity to prevent αsyn misfolding (Wang et al., 2018; Underwood et al., 2021). Our study here testing the impact of 14-3-3θ in Aβ models does not support the hypothesis that the protective effect of 14-3-3θ extends to Aβ mouse models. Indeed, the reduction of 14-3-3s in both human patients with AD and in AD mouse models (McFerrin et al., 2017; G. Xu et al., 2013) may instead point to this reduction in 14-3-3θ in AD human brains as a compensatory effect.

In conclusion, we examined the effect of 14-3-3θ on Aβ-induced toxicity in several AD models. While protective against Aβo-induced toxicity in cortical cultures, 14-3-3θ overexpression failed to rescue cognitive decline and pathologic changes in two APP mouse models. These findings do not support the idea of manipulating 14-3-3θ as a therapeutic approach for AD.

**Table 1:** Summary of statistical tests used

Figure	Experiment	*n*	Type of test	Statistical analysis
Figure 1*F*	Aβ toxicity assay, cortical culture, nTg vs 14-3-3θ	nTg = 6; 14-3-3θ = 7	Two-way ANOVA	Genotype: *F*_(1,33)_ = 20.28; *p* < 0.0001 Ab dose: *F*_(2,33)_ = 38.67; *p* < 0.0001 Interaction: *F*_(2,33)_ = 15.40, *p* < 0.0001 Sidak’s: nTg vs 14-3-3θ at 0 μm *p* = 0.2173, at 1 μm *p* = 0.0012, at 5 μm *p* < 0.0001
Figure 1*G*	Aβ toxicity assay, hippocampal culture, nTg vs 14-3-3θ	nTg = 6–7; 14-3-3θ = 7	Two-way ANOVA	Genotype: *F*_(1,35)_ = 5.801, *p* = 0.0214 Ab dose: *F*_(2,35)_ = 27.00, *p* < 0.0001 Interaction: *F*_(2,35)_ = 1.626, *p* = 0.2112 Sidak’s: nTg vs 14-3-3θ at 0 μm *p* = 0.0506, at 1 μm *p* > 0.9999, at 5 μm *p* = 0.3181
Figure 1*H*	Aβ toxicity assay, cortical culture, nTg vs difopein	*n* = 4	Two-way ANOVA	Genotype: *F*_(1,18)_ = 25.85, *p* < 0.0001 Ab dose: *F*_(2,18)_ = 42.68; *p* < 0.0001 Interaction: *F*_(2,18)_ = 9.481, *p* =0.0015 Sidak’s: nTg vs 14-3-3θ at 0 μm *p* = 0.9551, at 1 μm *p* = 0.0044, at 5 μm *p* < 0.0001
Figure 1*I*	Aβ toxicity assay, hippocampal culture, nTg vs difopein	*n* = 4	Two-way ANOVA	Genotype: *F*_(1,18)_ = 3.952, *p* = 0.0622 Ab dose: *F*_(2,18)_ = 16.47, *p* < 0.0001 Interaction: *F*_(2,18)_ = 0.6126, *p* = 0.5529 Sidak’s: nTg vs 14-3-3θ at 0 μm *p* = 0.9639, at 1 μm *p* = 0.1763, at 5 μm *p* = 0.6845
Figure 2*E*	J20, 2 MPI, open field distance traveled	nTg/GF*p* = 10; nTg/14-3-3θ = 10; J20/GF*p* = 9; J20/14-3-3θ = 8	Two-way ANOVA	Interaction: *F*_(1,33)_ = 3.746, *p* = 0.0615 APP genotype factor: *F*_(1,33)_ = 45.57, *p* < 0.001 AAV injection type factor: *F*_(1,33)_ = 5.910, *p* = 0.0207 Tukey’s: nTg/GFP vs J20/GFP, *p* = 0.0081; nTg/14-3-3θ vs J20/14-3-3θ, *p* < 0.0001; J20/GFP vs J20/14-3-3θ, *p* = 0.0279; nTg/GFP vs J20/GFP, *p* = 0.0081; nTg/14-3-3θ vs J20/GFP, *p* = 0.0184
Figure 2*F*	J20, 2 MPI, EPM percent time in open arm	nTg/GF*p* = 10; nTg/14-3-3θ = 10; J20/GF*p* = 9; J20/14-3-3θ = 8	Two-way ANOVA	Interaction: *F*_(1,33)_ = 0.8565, *p* = 0.3614 APP genotype factor: *F*_(1,33)_ = 2.451, *p* = 0.1270 AAV injection type factor: *F*_(1,33)_ = 0.8338, *p* = 0.3678
Figure 2*G*	J20, 2 MPI, MWM latency to platform	nTg/GF*p* = 10; nTg/14-3-3θ = 10; J20/GF*p* = 9; J20/14-3-3θ = 7	Three-way ANOVA	Time: *F*_(4,85)_ = 16.33, *p* < 0.0001 Genotype: *F*_(1,85)_ = 105.0, *p* < 0.0001 Virus: *F*_(1,79)_ = 2.544, *p* = 0.1147 Time × genotype: *F*_(4,85)_ = 3.840, *p* = 0.0064 Time × virus: *F*_(4,79)_ = 0.7289, *p* = 0.5749 Genotype × virus: *F*_(1,79)_ = 2.266, *p* = 0.1362 Time × genotype × virus: *F*_(4,79)_ = 2.713, *p* = 0.0357 Tukey’s: day 5 comparisons: nTg/GFP vs nTg/14-3-3θ, *p* > 0.9999; nTg/GFP vs J20/GFP, *p* = 0.1287; J20/GFP vs J20/14-3-3θ, *p* = 0.5494; nTg/GFP vs J20/14-3-3θ, *p* < 0.0001; nTg/14-3-3θ vs J20/GFP, *p* = 0.0794; ntg/14-3-3θ vs J20/14-3-3θ *p* < 0.0001
Figure 2*H*	J20, 2 MPI, MWM probe trial, time in target quadrant	nTg/GF*p* = 10; nTg/14-3-3θ = 10; J20/GF*p* = 9; J20/14-3-3θ = 7	Two-way ANOVA	Interaction: *F*_(1,32)_ = 4.622, *p* = 0.0392 APP genotype factor: *F*_(1,32)_ = 3.675, *p* = 0.0642 AAV injection type factor: *F*_(1,32)_ = 3.170, *p* = 0.5773 Tukey’s: nTg/14-3-3θ vs J20/14-3-3θ, *p* = 0.0426
Figure 2*I*	AAV-injected J20 survival curve	nTg/GF*p* = 7; nTg/14-3-3θ = 10; J20/GF*p* = 10; J20/14-3-3θ = 11	log-rank/Mantel–Cox	*p* = 0.201
Figure 2*J*	J20, 6 MPI, open field distance traveled	nTg/GF*p* = 9; nTg/14-3-3θ = 10; J20/GF*p* = 7; J20/14-3-3θ = 5	Two-way ANOVA	Interaction: *F*_(1,27) _= 0.7530, *p* = 0.3932 APP genotype factor: *F*_(1,27)_ = 40.84, *p* < 0.0001 AAV injection type factor: *F*_(1,27)_ = 0.1557, *p* = 0.9016 Tukey’s: nTg/GFP vs J20/GFP, *p* < 0.0001; nTg/14-3-3θ vs J20/14-3-3θ, *p* = 0.0044; nTg/GFP vs J20/14-3-3θ, *p* = 0.0015; nTg/14-3-3θ vs J20/GFP, *p* = 0.0002
Figure 2*K*	J20, 6 MPI, EPM percent time in open arm	nTg/GF*p* = 9; nTg/14-3-3θ = 10; J20/GF*p* = 7; J20/14-3-3θ = 5	Two-way ANOVA	Interaction: *F*_(1,27)_ = 0.3644, *p* = 0.5511 APP genotype factor: *F*_(1,27)_ = 4.821, *p* = 0.0369 AAV injection type factor: *F*_(1,27)_ = 0.1706, *p* = 0.6828
Figure 2*L*	J20, 6 MPI, MWM latency to platform	nTg/GF*p* = 9; nTg/14-3-3θ = 10; J20/GF*p* = 7; J20/14-3-3θ = 5	Three-way ANOVA	Time: *F*_(4,75)_ = 12.86, *p* < 0.0001 Genotype: *F*_(1,75)_ = 46.69, *p* < 0.0001 Virus: *F*_(1,60)_ = 6.839, *p* = 0.0113 Time × genotype: *F*_(4,75)_ = 0.5912, *p* = 0.6700 Time × virus: *F*_(4,60)_ = 0.6958, *p* = 0.5978 Genotype × virus: *F*_(1,60)_ = 0.3838, *p* = 0.5379 Time × genotype × virus: *F*_(4,60)_ = 0.2333, *p* = 0.9186 Tukey’s: day 5 comparisons: nTg/GFP vs nTg/14-3-3θ, *p* > 0.9999; nTg/GFP vs J20/GFP, *p* = 0.1562; J20/GFP vs J20/14-3-3θ, *p* > 0.9999; nTg/GFP vs J20/14-3-3θ, *p* = 0.3373; nTg/14-3-3θ vs J20/GFP, *p* = 0.3086; ntg/14-3-3θ vs J20/14-3-3θ *p* = 0.5425
Figure 3*C*	APP KI, 8–9 months, open field distance traveled	nTg = 10; APP KI = 16; APP KI/14-3-3θ = 11	One-way ANOVA	*F*_(2,34)_ = 0.9122, *p* = 0.4112
Figure 3*D*	APP KI, 8–9 months, EPM percent time in open arm	nTg = 10; APP KI = 15; APP KI/14-3-3θ = 9	One-way ANOVA	*F*_(2,31)_ = 11.17, *p* = 0.0002 Tukey’s: APP KI/14-3-3θ vs APP KI, *p* = 0.0001; APP KI/14-3-3θ- vs nTg, 0.0175
Figure 3*E*	APP KI, 8–9 months, passive avoidance latency to dark side, D2	nTg = 10; APP KI = 14; APP KI/14-3-3θ = 8	One-way ANOVA	*F*_(2,29)_ = 0.1705, *p* = 0.8441
Figure 4*A*	J20 hippocampal Aβ plaque load	J20/GF*p* = 6; J20/14-3-3θ = 5	Unpaired, two-tailed, *t* test	*t* = 0.09254, df = 9; *p* = 0.9238
Figure 4*B*	APP KI hippocampal Aβ plaque load	APP KI = 16; APP KI/14-3-3θ = 10	Unpaired, two-tailed, *t* test	*t* = 1.038, df = 24; *p* = 0.3084
Figure 4*C*	APP KI cortical Aβ plaque load	APP KI = 15; APP KI/14-3-3θ = 10	Unpaired, two-tailed, *t* test	*t* = 1.475, df = 23; *p* = 0.1538
Figure 5*A*	J20 hippocampal synapsin immunoreactivity	nTg/GF*p* = 8; nTg/14-3-3θ = 9; J20/GF*p* = 5; J20/14-3-3θ = 5	Two-way ANOVA	Interaction: *F*_(1,23)_ = 0.3085, *p* = 0.5839 APP genotype factor: *F*_(1,23)_ = 0.1737, *p* = 0.6807 AAV injection type factor: *F*_(1,23)_ = 0.7492, *p* = 0.3957
Figure 5*B*	APP KI hippocampal synapsin immunoreactivity	nTg = 9; APP KI = 16; APP KI/14-3-3θ = 9	One-way ANOVA	*F*_(2,31)_ = 0.4169, *p* = 0.6627

## Synthesis

Reviewing Editor: Karl Herrup, University of Pittsburgh School of Medicine Decisions are customarily a result of the Reviewing Editor and the peer reviewers coming together and discussing their recommendations until a consensus is reached. When revisions are invited, a fact-based synthesis statement explaining their decision and outlining what is needed to prepare a revision will be listed below. The following reviewer(s) agreed to reveal their identity: Zoltan Simandi, Jie Zhang.

The revised manuscript is significantly improved from the original submission. It remains disappointing that the in vivo studies were negative, but the explanation offered for the discordance is plausible. Only a few minor changes are needed before a final acceptance

1. The authors report that 14-3-3 AAV injection in hippocampus significantly shortened the lifespan of the J20 mice. It would help the reader interpret the findings if they were to provide the mortality data for the APP-KI and APP-KI/14-3-3 mice.

2. Based on the amyloid staining in Figure 4B, the amyloid plaque burden is obviously increased in hippocampus of APP KI/14-3-3 mice compared with APP KI mice. While, the variation in statistics analysis is very big, the authors should add the sample numbers or selected more representative images.

3. To better document the effects of the experimental manipulation on synaptic integrity, immunostaing for synapses should be provided. Any number of agents could be used, for example synapsin or synaptophysin/PSD95 double staining. This should be done in both J20 and APP KI mice brain.

## Author Response

Dear Dr. Herrup,

Enclosed is a revised manuscript entitled “14-3-3θ does not protect against behavioral or pathological deficits in Alzheimer’s disease mouse models” that we would like to resubmit for publication in eNeuro. All authors have read and approved this manuscript.

We appreciate the comments by the reviewers of our revised manuscript. Overall, the reviewers found the work significantly improved from the initial submission and require a few minor changes before acceptance. A point-by-point response detailing changes to the manuscript is below:

1. To help clarify that the 14A3 mice did not have the same premature mortality that we saw with the J20 mice, we have included a survival curve for the 14A3 mice (Figure 3B). This should make it easier for the readers to interpret the data.

2. As noted by the reviewer, we do not see a difference in the beta amyloid burden between the APP-KI and APP-KI/14-3-3 mice, but the representative images that we had chosen for the hippocampus of these mice showed increased plaque load in the APP-KI/14-3-3θ image. We have selected better representative images to reflect our findings more accurately.

3. The reviewer asks us to provide better documentation of the effects of the experimental manipulation on synaptic integrity. We had previously quantified and provided representative images of synaptic density based on synapsin staining (Fig. 5), but to validate synaptic integrity, we have added a co-stain using a presynaptic marker VGLUT1 and a postsynaptic marker NMDAR 2A (Fig. 5).

We feel that our manuscript is stronger by addressing the reviewers’ concerns. We hope that our revisions have adequately addressed the reviewers’ concerns and meet the criteria for publication in eNeuro.
